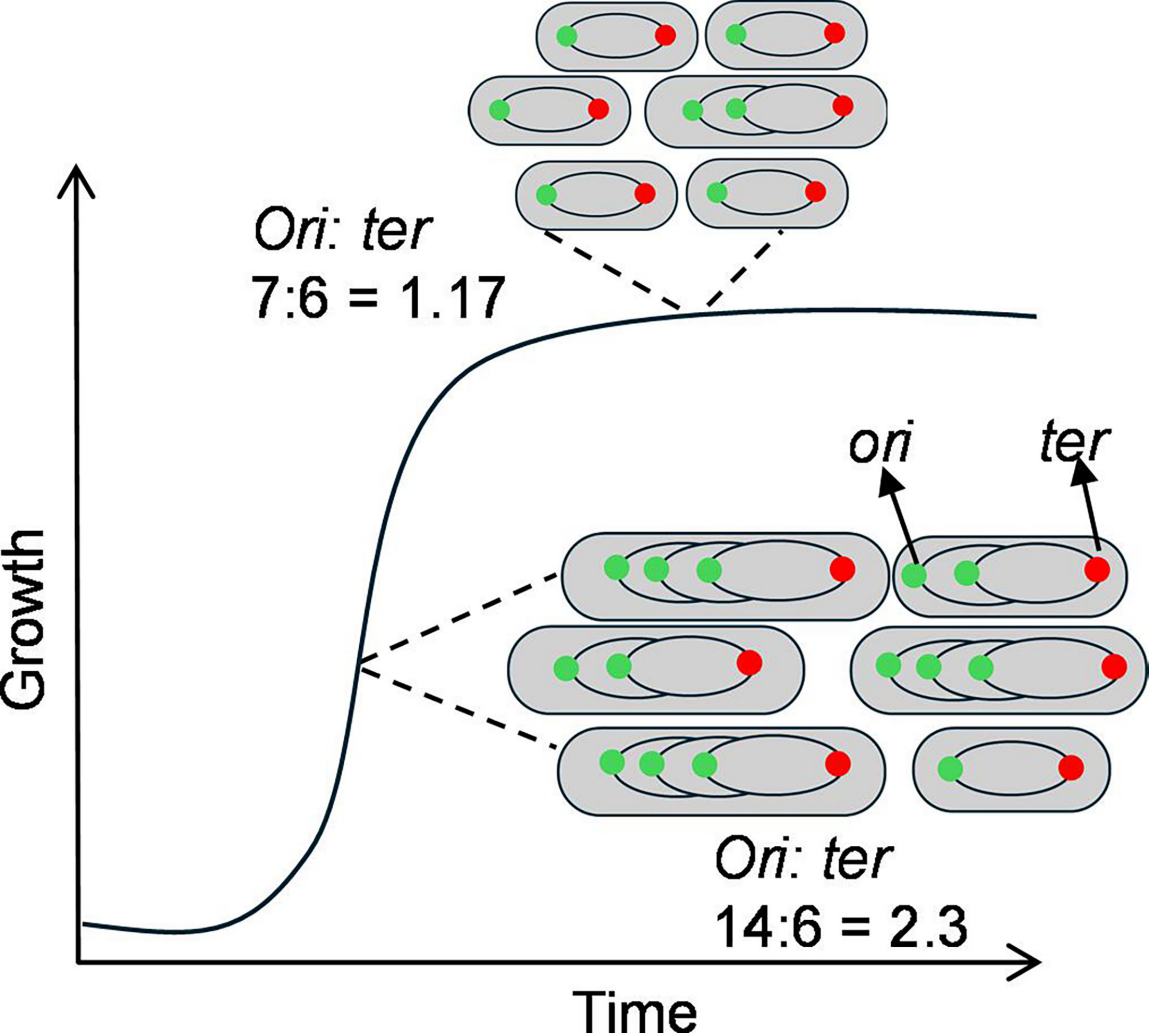# Articles of Significant Interest in This Issue

**DOI:** 10.1128/aem.01592-25

**Published:** 2025-08-20

## THE SQUID LIGHT ORGAN GETS A FACELIFT

Imes et al. (e00001-25) report similar phenotypes in the symbiotic light organs of the well-known Hawaiian bobtail squid and the hummingbird variety that is robust to laboratory husbandry and amenable to genetic manipulation. This makes the hummingbird bobtail squid a strong symbiosis model for future functional studies.


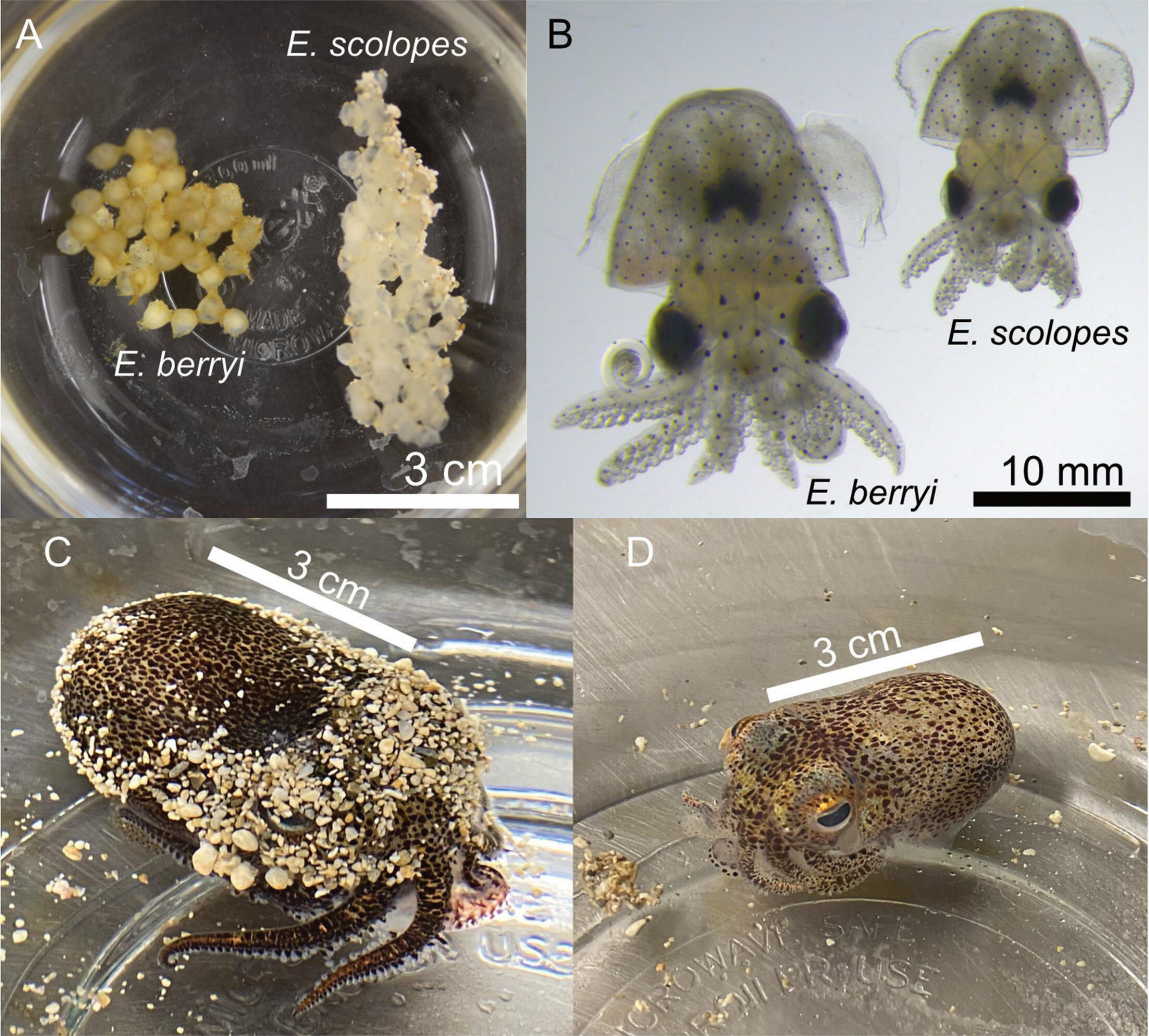


## *MANGROVIBACTER* RESEARCH IN REVIEW

Timely review by Chin et al. (e02479-24) of an understudied yet ubiquitous bacterial genus (*Mangrovibacter*) that is key to bioremediation and biodegradation applications, as well as in the enhancement of plant growth and food processing.


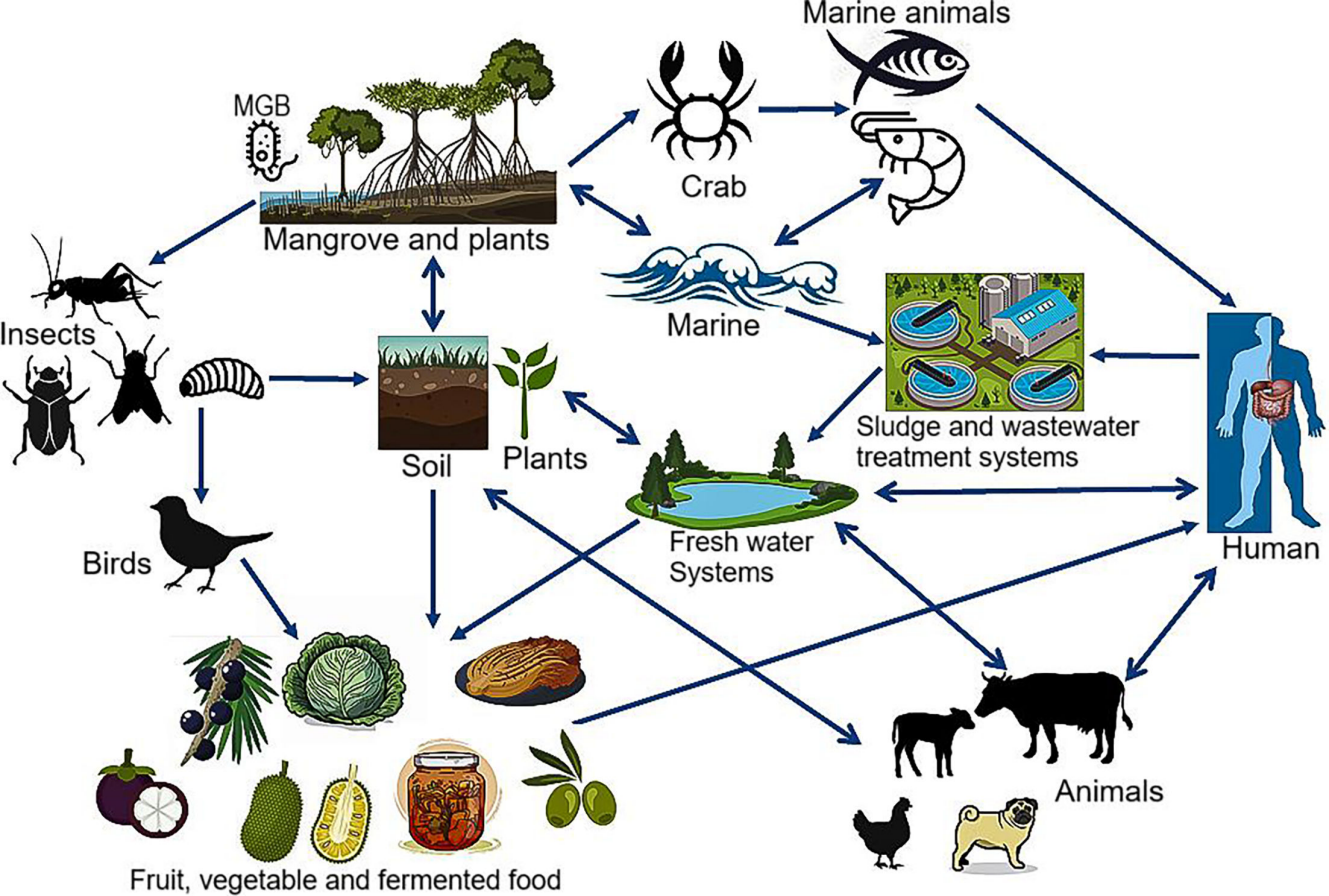


## THE MICROBIAL LEGACY OF MINING

Past mining operations have left ecosystems of waste with microbial communities adapted to the unique geochemistry of their environment. Best et al. (e00434-25) show that these microbes represent important resources for bioremediation and bioreclamation of valuable elements.


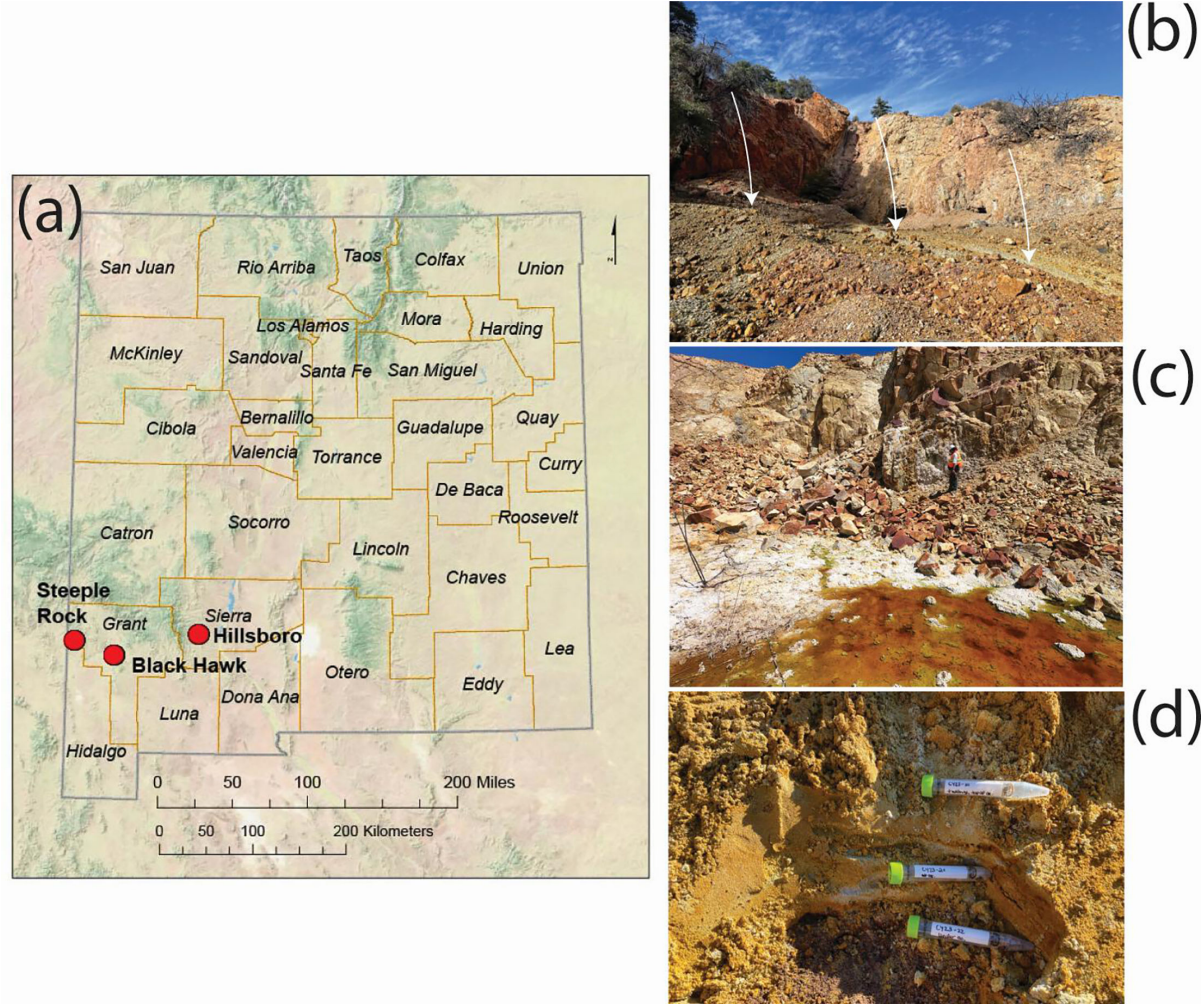


## AMICOUMACINS TO THE CITRUS TREE RESCUE!

In this study by Vieira and colleagues (e00869-25), amicoumacins produced by a *Bacillus* isolate show promise as biocontrol agents against the Huanglongbing disease that devastates citrus trees worldwide.


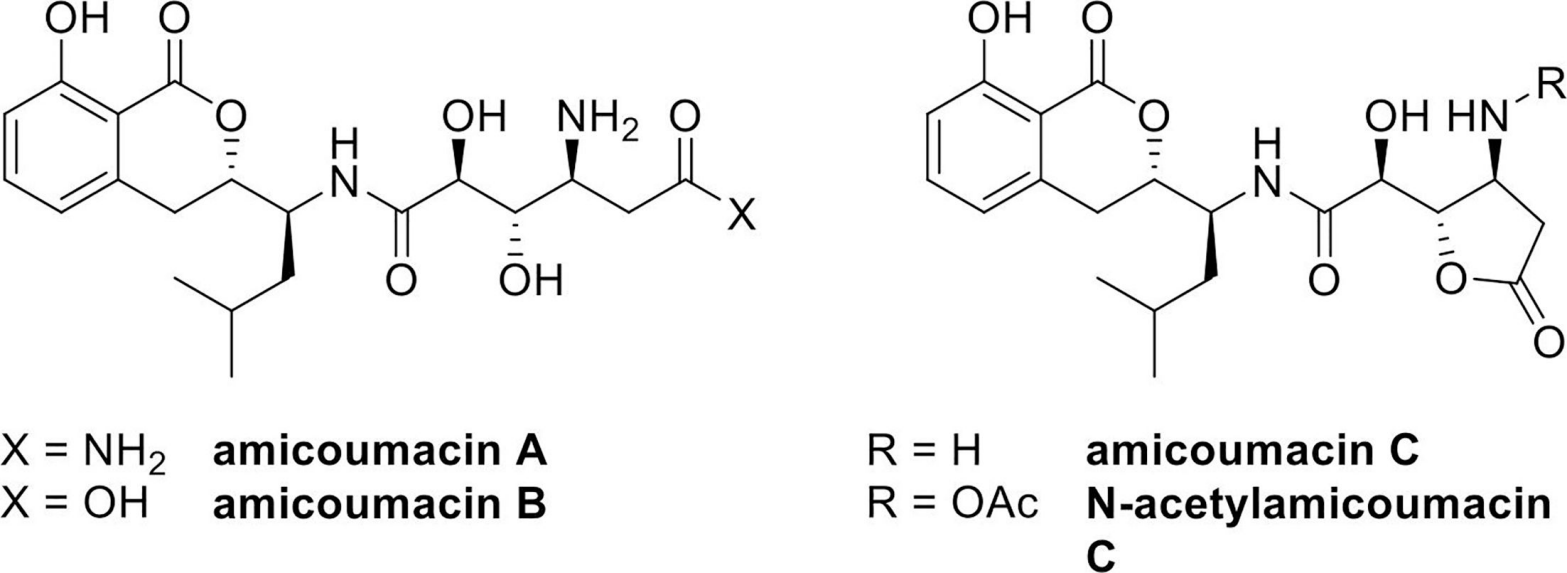


## AN EXPANDED VIEW OF AEROBIC METHANOTROPHS

A new methanotroph in the *Actinomycetota* has broad pH tolerance and high resistance to ammonia, a ubiquitous competitive inhibitor of methanotrophy. These findings by Kambara et al. (e00796-25) challenge current knowledge of methanotrophic physiology and the ecological niches that could serve as methane sinks.


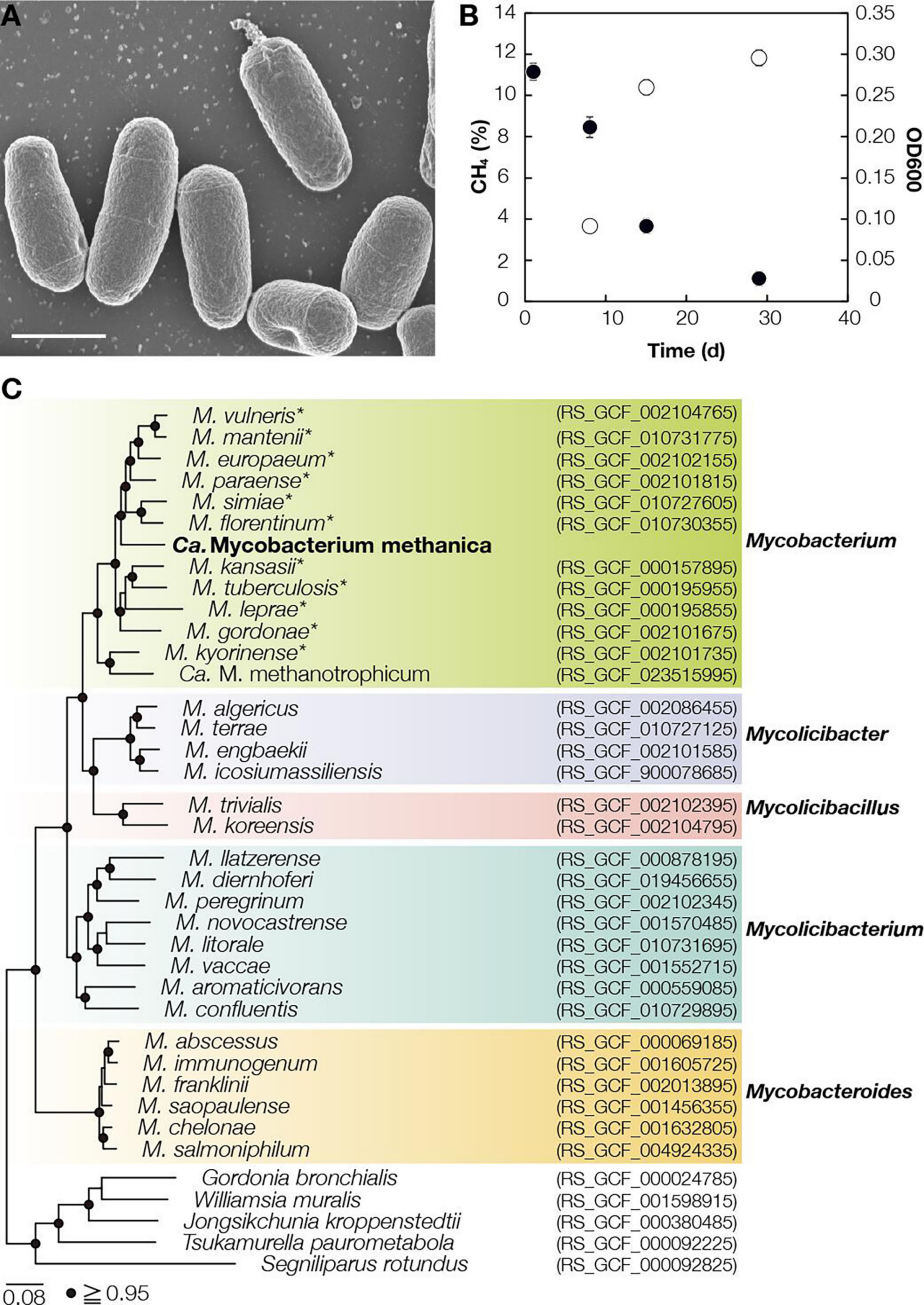


## FUNGAL AEROSOL DETECTION MEETS ELISA

Pogner and team (e00163-25) share a new ELISA method for the identification of airborne fungal spores that greatly improves bioaerosol surveillance and the detection of disease-causing agents.


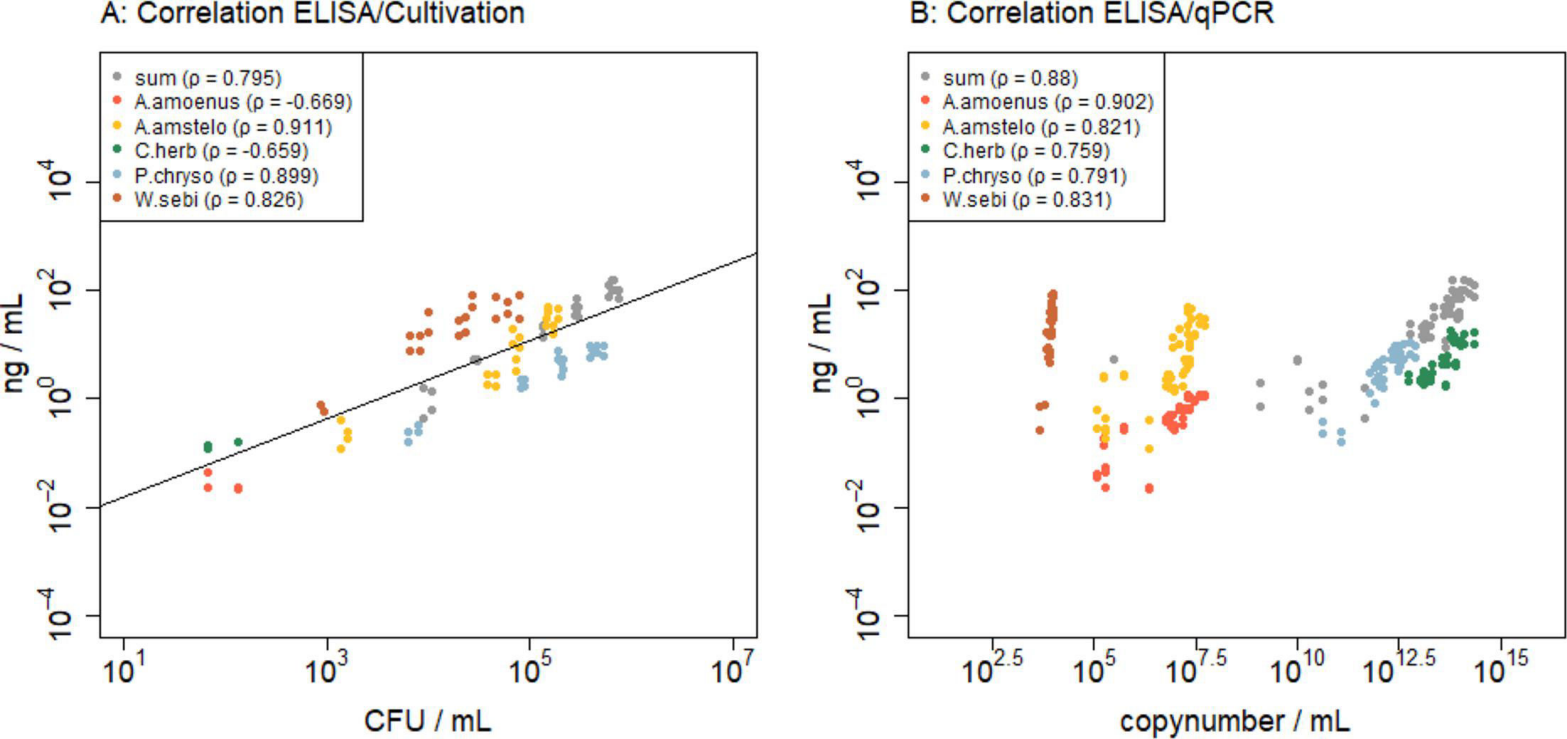


## LIPOPEPTIDES FOR CROP PROTECTION

Caravaca-Fuentes et al. (e00734-25) share that bactericidal lipopeptides with specificity for the lipopolysaccharide of *Xylella fastidiosa* afford opportunities for control of this phytopathogen and protection of crops of economic importance.


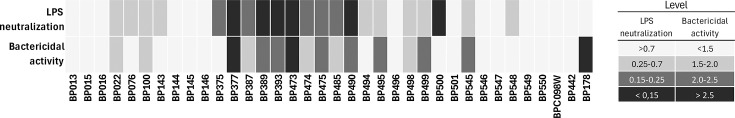


## PATHOGENS CAUGHT IN THE ACT OF INFECTION

A PCR-based method by Paudel and colleagues (e02382-24) simplifies measurements of bacterial growth rates in the host to more accurately assess the success of pathogens in establishing infections.